# Network Code DGNSS Positioning for Faster L1–L5 GPS Ambiguity Initialization

**DOI:** 10.3390/s20195671

**Published:** 2020-10-04

**Authors:** Mieczysław Bakuła, Marcin Uradziński, Kamil Krasuski

**Affiliations:** 1 Faculty of Geoengineering, University of Warmia and Mazury, 10-719 Olsztyn, Poland; marcin.uradzinski@uwm.edu.pl; 2 Institute of Navigation, Military University of Aviation, 08-521 Dęblin, Poland; k.krasuski@law.mil.pl

**Keywords:** DGPS, DGNSS, differential positioning, Kalman filter, PREFMAR

## Abstract

This paper presents DGNSS network code positioning using permanent geodetic networks, commonly used in GNSS measurements. Using several reference stations at the same time allows for the independent control of GNSS positioning and facilitates the more realistic estimation of accuracy. Test calculations were made on the basis of real GPS data, using one TRIMBLE mobile receiver and four nearest reference stations of the ASG-EUPOS geodetic system. In addition, DGNSS positioning computational simulations were performed for a case where one mobile GNSS receiver would be able to be used with two (e.g., GPS + Galileo or GPS + GLONASS) or four different positioning systems and different GNSS reference station systems at the same time. To reduce the deviations of the DGPS positioning from a true value, the Kalman filtering for horizontal coordinates and vertical ones was used. The result shows a significant improvement in DGPS positioning accuracy. Based on the numerical analysis carried out, it can be seen that when four GNSS systems are used, it is possible to achieve a DGNSS accuracy of 0.1 m and 0.2 m for horizontal and height coordinates, respectively, using only code measurements. Additionally, the paper presents the impact of the DGNSS code positioning accuracy on the effectiveness of determining ambiguities of phase observations on individual measurement epochs, using the L1–L5 observations of the GPS system and the precise and fast method of ambiguity resolution (PREFMAR). The developed DGNSS positioning methodology can be applied for reliable GNSS navigation using at least two independent GNSS systems.

## 1. Introduction

Differential global positioning system (DGPS) measurements have been known since the beginning of GPS technology. In general, this positioning technology is based on the use of only code receivers, mainly civil code (CA), which is available in every GPS receiver. In differential positioning, code-based DGPS is also widely used not only in navigation, but also in surveying and mapping [1,2,3,4,5]. It has the advantages of simplified algorithms and preferable precision. Although the accuracy of code measurements is incomparably worse than phase measurements, paradoxically, code measurements are nowadays indispensable in every GNSS receiver. Even when we get centimeter and even millimeter accuracy, at some stage, the code measurements must always take part in determining the exact coordinates, and more importantly, the phase positioning efficiency depends on the code measurements [6]. Therefore, as there is currently little research on the effectiveness of DGNSS positioning in favor of real time kinematic (RTK) phase positioning, DGNSS positioning may play an important role in the future. This is due to the expansion of satellite positioning systems and the possibility of independent and reliable GNSS positioning, especially for precise and reliable navigation.

There are many different studies on DGNSS positioning algorithms. Some have been mainly focused on the combination of using one satellite for each system in relative positioning and one receiver clock parameter for one system in single-point positioning [7,8]. In the case of a multi-system solution, an inter-system bias (ISB) is usually considered. Based on the presence of ISB, the regular DGNSS model should use separate clock parameters for each system to establish the precision of positioning results and improve the performance of the DGNSS method. Such a real time BeiDou/GLONASS/Galileo model accounting for ISB was proposed by [9]. Additionally, one should pay attention to the use of GNSS code biases between signals while modeling the ionosphere. The source of code biases along with their effects on GNSS positioning, and their estimation, can be found in various research works [10,11,12]. Differential code bias (DCB) of a GNSS receiver is usually precisely estimated and corrected while using the GNSS ground network. It is based on the characterization of the global ionosphere along with the global ionospheric maps. Following other researchers, differential code bias can be also estimated using a recursive method along with the selection of an individual reference station [13]. Among quoted techniques, the DGNSS technique using pseudorange correction (PRC) has also been widely used in a number of research fields improving real-time positioning accuracy in cheap receivers. Positioning accuracy obtained by researchers using predicted PRC demonstrated that for DGPS and DBeiDou horizontal errors were at the one meter level, which definitely would be very useful to continue DGNSS positioning during correction data outages [14]. The estimation and analysis of code biases is also essential for ambiguity resolution when depending on the pseudorange method. This is well known for the double differencing method and also for the undifferenced occurrence [15]. For this purpose, the use of observable specific signal biases (OSB) can be chosen as an alternative analysis of code biases [16].

Concerning the usefulness of GNSS technology, it should be mentioned that in recent years, there has also been a huge increase in the interest in positioning using mobile phones. This means that the DGNSS technique would be implemented using smartphones [17,18]. Nowadays, the modern smartphones or mass-market portable mobile receivers with built-in GNSS chipsets can reach very impressive positioning quality, which became a practical tool in the spreading of location-based applications. Since 2016, scientists have been focusing on the usefulness of GNSS observations derived from mobile phones [19,20,21,22,23]. Application programming interface (API) is well known as a set of predefined functions for developing custom applications to interface with the GNSS chipset for obtaining not only pseudorange information but also carrier phase observations. Today, for navigational applications, the regular positioning accuracy achieved in smartphone ranges from a few meters to tens of meters (under difficult conditions). This situation occurs due to pseudoranges which are mostly used for low-precision positioning. DGNSS users may obtain positioning accuracy up to 1 m or better, which is satisfactory for various applications, such as intelligent transportation systems (ITS) or as a technique for the recovery of aircraft position, where the DGNSS system with additional devices is required for control and safety systems [24,25,26,27].

In our work, research was carried out on the use of DGNSS technology when we have one, two or as many as four reference station systems at our disposal. The simulation studies used the real data of a GPS-only system to prevent (at this stage of analysis) the errors associated with the integration of GNSS systems. However, it can be assumed that the DGPS positioning accuracy is at a similar level as the positioning accuracy of DGLONASS, DGalileo or DBeiDou, in the situation where these systems are fully configured. Considering this solution working for professional receivers, the GNSS code observations obtained from recent mobile phones will be also considered by us for analysis in the upcoming project. The work consists of two main parts, in which the first part presents DGNSS positioning accuracy analysis with two and four independent GNSS systems. The second part discusses the influence of DGNSS positioning accuracy on the effectiveness of ambiguity determination for individual observation epochs, using the PREFMAR method, for a baseline with a length of 4 km.

## 2. DGPS Positioning Based On Least Squares Method and Two Scenarios of Network DGNSS Positioning

Generally, in DGPS positioning, we only use code observations on the L1 frequency. We can therefore write the following observation Equation for each satellite [28]:

(1) $$P\left(t\right)\;=\;\rho \left(t\right)+cdt\left(t\right)-cdT\left(t\right)+d_{ION}\left(t\right)+d_{TROP}\left(t\right)+d_{EPHEM}\left(t\right)+d_{P}\left(t\right)$$

where P(t) is the measured pseudorange, ρ(t) is the true receiver-to-satellite geometric range, c is the speed of light, dt(t) is the satellite clock error, dT(t) is the receiver clock error, $d_{ION}\left(t\right)$ is the ionospheric delay error, $d_{TROP}\left(t\right)$ is the tropospheric delay error, $d_{EPHEM}\left(t\right)$ is the satellite ephemeris error, and $d_{P}\left(t\right)$ represents other pseudorange errors, such as multipath, interchannel receiver biases, thermal noise, receiver and satellite hardware delay, as well as pseudorange measurement noise.

The pseudorange correction (PRC) for a satellite i at the epoch (t) is calculated by the Equation [29]:

(2) $$PRC^{i}\left(t\right)\;=\;\rho_{REF}^{i}\left(t\right)-P_{REF}^{i}\left(t\right)$$

where:

(3) $$\rho_{REF}^{i}\left(t\right)\;=\;\sqrt{{\left(X^{s}\left(t\right)-X_{REF}\right)}^{2}+{\left(Y^{s}\left(t\right)-Y_{REF}\right)}^{2}+{\left(Z^{s}\left(t\right)-Z_{REF}\right)}^{2}}$$

The determined values $PRC^{i}\left(t\right)$ for some satellites are included in the mathematical model of autonomous positioning in a GNSS receiver, the coordinates of which should be determined in the reference stations’ frame. Therefore, for satellites *i*, *j*, *k*, *l*, and the designated station M, we can use a system of Equations in the form of a matrix [29]:

(4) L = AX

where L and A matrixes are as follows:

(5) $$L\;=\;\left[\begin{array}{c} \begin{array}{c} P_{M}^{i}\left(t\right)-\rho_{M,0}^{i}\left(t\right)+cdt^{i}\left(t\right)+PRC_{REF}^{i}\left(t\right)\\ P_{M}^{j}\left(t\right)-\rho_{M,0}^{j}\left(t\right)+cdt^{j}\left(t\right)+PRC_{REF}^{j}\left(t\right) \end{array}\\ \begin{array}{c} P_{M}^{k}\left(t\right)-\rho_{M,0}^{k}\left(t\right)+cdt^{k}\left(t\right)+PRC_{REF}^{k}\left(t\right)\\ P_{M}^{l}\left(t\right)-\rho_{M,0}^{l}\left(t\right)+cdt^{l}\left(t\right)+PRC_{REF}^{l}\left(t\right) \end{array} \end{array}\right]$$

(6) $$A=\left[\begin{array}{c} \begin{array}{cccc} -(X^{i}(t)-X_{M,0}){\left(\rho_{M,0}^{t}(t)\right)}^{-1} & -(Y^{i}(t)-Y_{M,0}){\left(\rho_{M,0}^{t}(t)\right)}^{-1} & -(Z^{i}(t)-Z_{M,0}){\left(\rho_{M,0}^{t}(t)\right)}^{-1} & c \end{array}\\ \begin{array}{cccc} -(X^{j}(t)-X_{M,0}){\left(\rho_{M,0}^{j}(t)\right)}^{-1} & -(Y^{j}(t)-Y_{M,0}){\left(\rho_{M,0}^{j}(t)\right)}^{-1} & -(Z^{j}(t)-Z_{M,0}){\left(\rho_{M,0}^{j}(t)\right)}^{-1} & c \end{array}\\ \begin{array}{cccc} -(X^{k}(t)-X_{M,0}){\left(\rho_{M,0}^{k}(t)\right)}^{-1} & -(Y^{k}(t)-Y_{M,0}){\left(\rho_{M,0}^{k}(t)\right)}^{-1} & -(Z^{k}(t)-Z_{M,0}){\left(\rho_{M,0}^{k}(t)\right)}^{-1} & c \end{array}\\ \begin{array}{cccc} -(X^{l}(t)-X_{M,0}){\left(\rho_{M,0}^{l}(t)\right)}^{-1} & -(Y^{l}(t)-Y_{M,0}){\left(\rho_{M,0}^{l}(t)\right)}^{-1} & -(Z^{l}(t)-Z_{M,0}){\left(\rho_{M,0}^{l}(t)\right)}^{-1} & c \end{array} \end{array}\right]$$

(7) $$X\;=\;\left[\begin{array}{c} \begin{array}{c} dX_{M}\left(t\right)\\ dY_{M}\left(t\right) \end{array}\\ \begin{array}{c} dZ_{M}\left(t\right)\\ dT_{m}\left(t\right) \end{array} \end{array}\right]$$

and the solution with the least squares method is:

(8) $$X\;=\;{\left(A^{T}PA\right)}^{-1}A^{T}PL,$$

where P is a unit diagonal weight matrix.

After determining the coordinates in the ETRF2000 system, based on Equation (8), the coordinates were transformed to the PL2000 planar system, obtaining the N (northing) and E (easting) coordinates, while the ellipsoidal height H was obtained by converting the Cartesian coordinates (XYZ) to BLH (Latitude, Longitude, and Ellipsoid Height) geodetic coordinates. In further analysis, the coordinates in NEH format were used, for which the calculations and analysis of DGNSS network positioning were carried out, designed for two scenarios:

Scenario no. I:

(1) Independent calculation of the DGPS position for the mobile receiver M using four reference stations of the ASG-EUPOS system and using the least squares method (LS); that is, the calculation for each observation epoch (t) of the coordinates of the measurement point M: $N_{M,REF}^{LS}\left(t\right)$, $E_{M,REF}^{LS}\left(t\right)$, $H_{M,REF}^{LS}\left(t\right)$;
(2) Then, calculate the average position as a DGPS network solution based on four reference stations determined in point (1):
  (9) $$\left[\begin{array}{c} N_{M}^{LS}(t)\\ E_{M}^{LS}(t)\\ H_{M}^{LS}(t) \end{array}\right]=4^{-1}\left[\begin{array}{c} N_{M,REF1}^{LS}(t)+N_{M,REF2}^{LS}(t)+N_{M,REF3}^{LS}(t)+N_{M,REF4}^{LS}(t)\\ E_{M,REF1}^{LS}(t)+E_{M,REF2}^{LS}(t)+E_{M,REF3}^{LS}(t)+E_{M,REF4}^{LS}(t)\\ H_{M,REF1}^{LS}(t)+H_{M,REF2}^{LS}(t)+H_{M,REF3}^{LS}(t)+H_{M,REF4}^{LS}(t) \end{array}\right]$$

Scenario no. II:

(1) Independent computation of the DGPS position for the rover using four reference stations, using the least squares method, and then applying Kalman filtering (KF) to the obtained LS coordinates, i.e., obtaining for each observation epoch (t) the coordinates of the measurement point M: $N_{M,REF}^{LS,KF}\;\left(t\right)$, $E_{M,REF\;}^{LS,KF}\left(t\right)$, $H_{M,REF}^{LS,KF}\;\left(t\right)$;
(2) Position calculation for a DGPS network solution based on the four reference stations specified in point (3):
  (10) $$\left[\begin{array}{c} N_{M}^{LS,KF}(t)\\ E_{M}^{LS,KF}(t)\\ H_{M}^{LS,KF}(t) \end{array}\right]=4^{-1}\left[\begin{array}{c} N_{M,REF1}^{LS,KF}(t)+N_{M,REF2}^{LS,KF}(t)+N_{M,REF3}^{LS,KF}(t)+N_{M,REF4}^{LS,KF}(t)\\ E_{M,REF1}^{LS,KF}(t)+E_{M,REF2}^{LS,KF}(t)+E_{M,REF3}^{LS,KF}(t)+E_{M,REF4}^{LS,KF}(t)\\ H_{M,REF1}^{LS,KF}(t)+H_{M,REF2}^{LS,KF}(t)+H_{M,REF3}^{LS,KF}(t)+H_{M,REF4}^{LS,KF}(t) \end{array}\right]$$

All the calculations presented in this paper were performed using our own software, both for DGPS positioning, using the least squares method [30] as well as our own algorithms using Kalman filtering to smooth the obtained coordinates from the LS method, which were also used for effective EGNOS positioning [31]. The noise of the observation model (for N, E, H) in the Kalman filtering was assumed at the level of 1.75 m, but the accuracy of the kinematic model in the Kalman filtering was set at 0.1 m for the north, east and height coordinates. The use of Kalman filtering significantly reduces the standard deviation of code GNSS positioning [32,33].

## 3. Description of Measurements and Numerical Analysis

**Figure 1:**
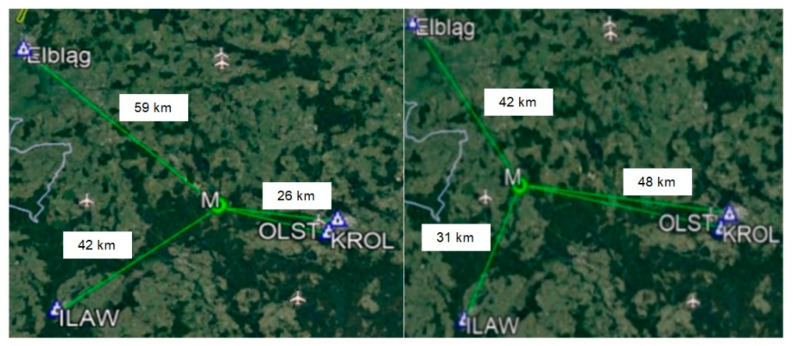
Arrangement of the reference stations of the ASG-EUPOS network during the differential global positioning system (DGPS) test measurements.

GPS test measurements were conducted on two different points, located between four reference stations: ELBL (Elbląg), OLST, KROL and ILAW (Figure 1). The receiver TRIMBLE SPS centered over the observation point (M), recorded GPS data with a measurement interval of 1 s.

**Figure 2:**
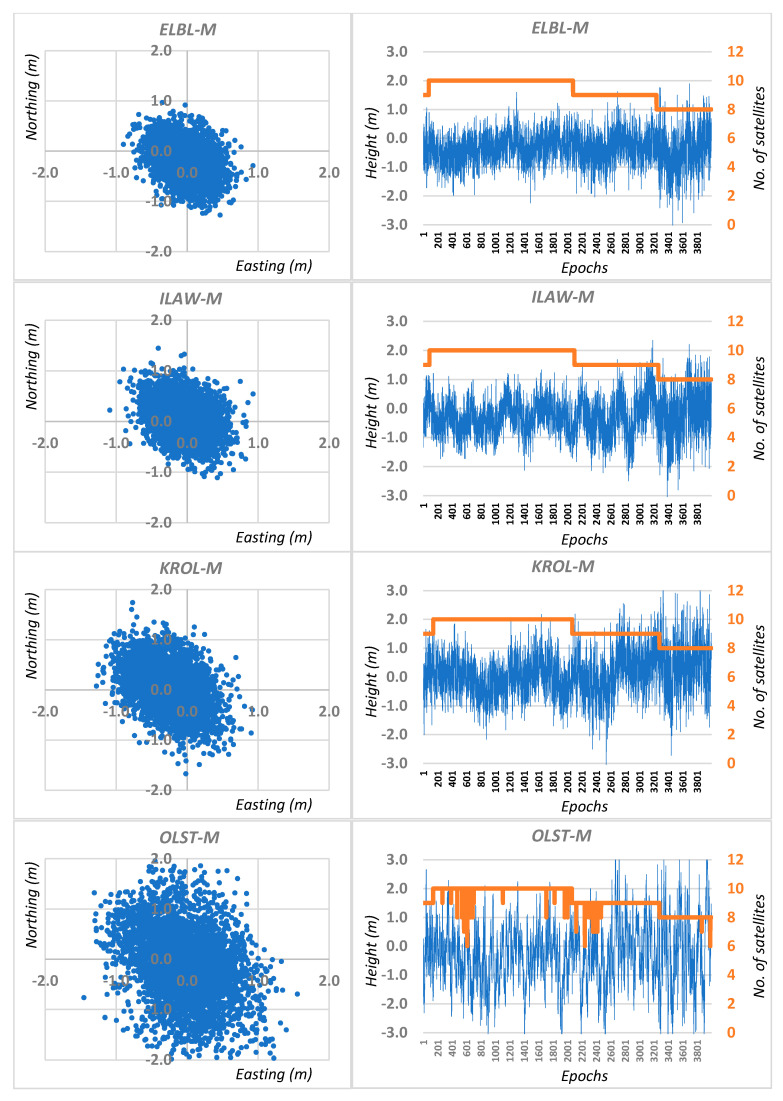
Real errors of single epoch traditional DGPS positioning based on least squares solutions for session I.

**Figure 3:**
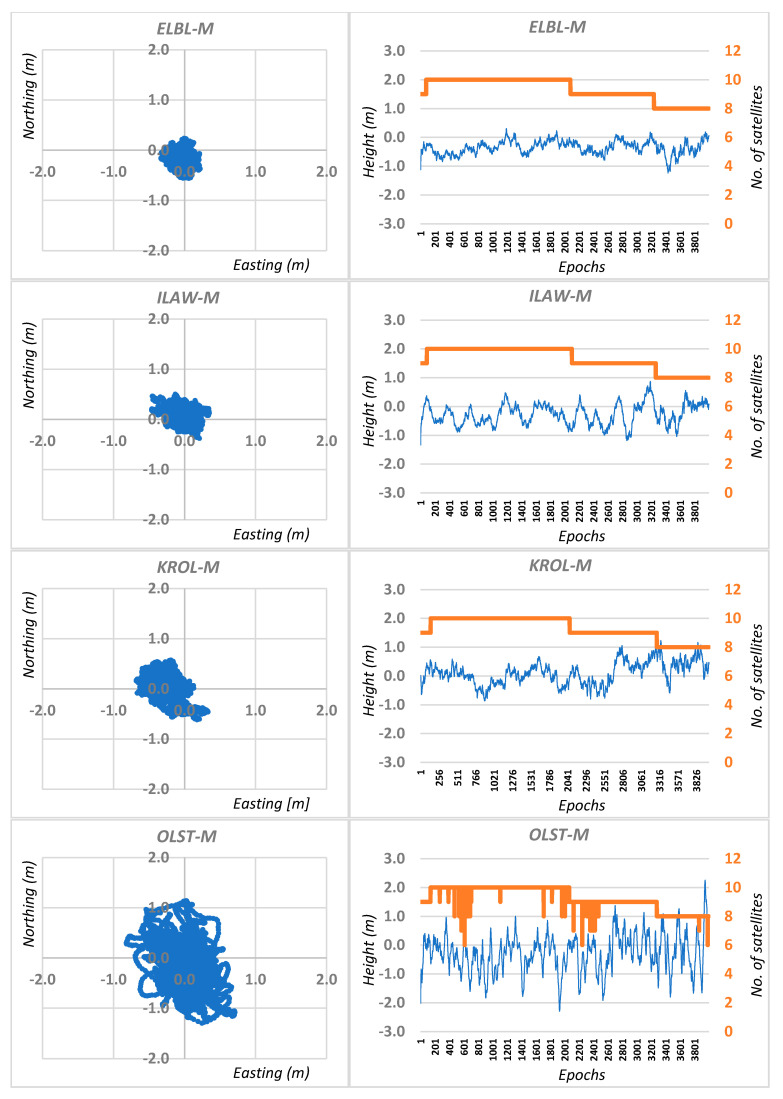
Real errors of single epoch DGPS positioning based on least squares solutions and Kalman filtering for session I.

Four thousand consecutive epochs were recorded on each point. For each measurement session, independent DGPS calculations were made for the M mobile receiver using the four closest reference stations located around the observation point. Therefore, four independent DGPS solutions based on the least squares method (LS) were obtained for each session: ELBL-M; ILAW-M; KROL-M and OLST-M (Figure 2). In addition, the Kalman filter (KF) was used for each solution (Figure 3).

**Figure 4:**
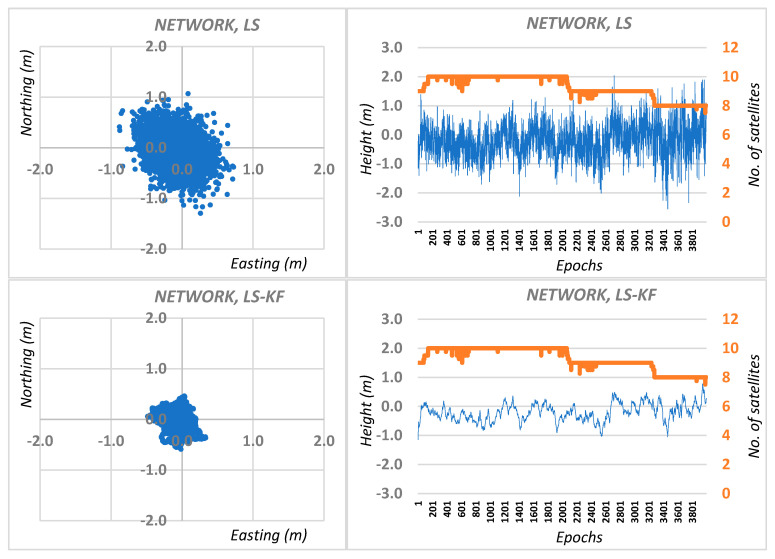
Real errors of network DGPS positioning based on least squares solutions and Kalman filtering (KF) for session I.

Then, network calculations of the N, E, H coordinates for M station were made as the arithmetic mean of solutions from different reference stations, both for traditional DGPS positioning and DGPS positioning using the Kalman filter. Graphic results are shown in Figure 4 for session I, while the average RMS network positioning errors for session I are shown in Table 1.

**Figure 5:**
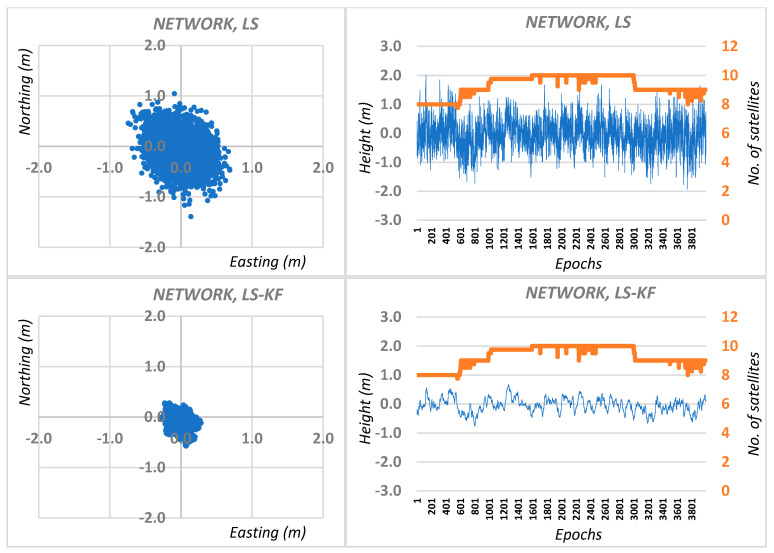
Real errors of network DGPS positioning based on least squares solutions and Kalman filtering, for session II.

Identical calculations were made for session II, where the observation point was at different locations and at a different observation time. The graphic results of network positioning for session II are shown in Figure 5, while the average RMS errors are presented in Table 2. Similar RMS errors were obtained for both session I and session II, i.e., for coordinate N the average DGMS network positioning RMS error was 0.232 and 0.217 m, for the E coordinate was 0.313 and 0.304 m, respectively, and for the heights 0.562 and 0.523 m. In the case of additional Kalman filtering, the average RMS errors were reduced by approximately 50%, and were as follows: for coordinate N: 0.131 and 0.100 m; for coordinate E, 0.167 and 0.135 m, and for coordinate H: 0.299 and 0.241 m.

**Figure 6:**
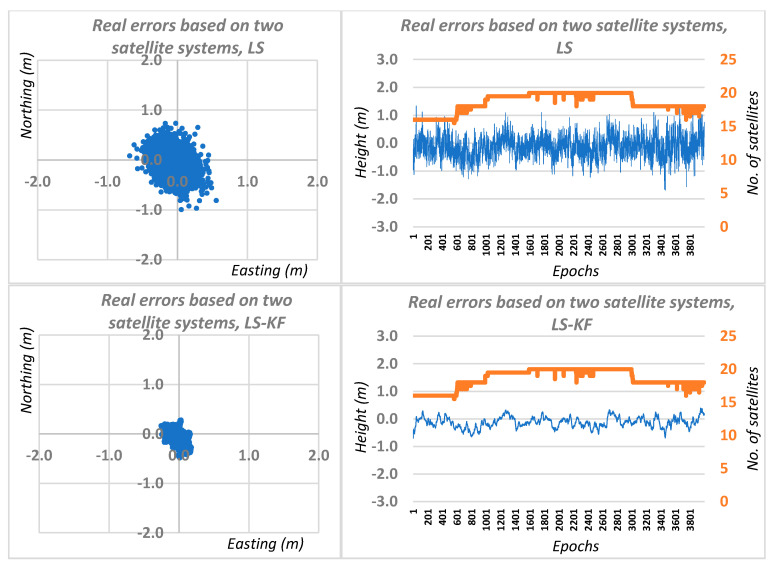
Real errors of the simulated network DGNSS positioning based on least squares solutions and Kalman filtering for two GNSS systems.

**Figure 7:**
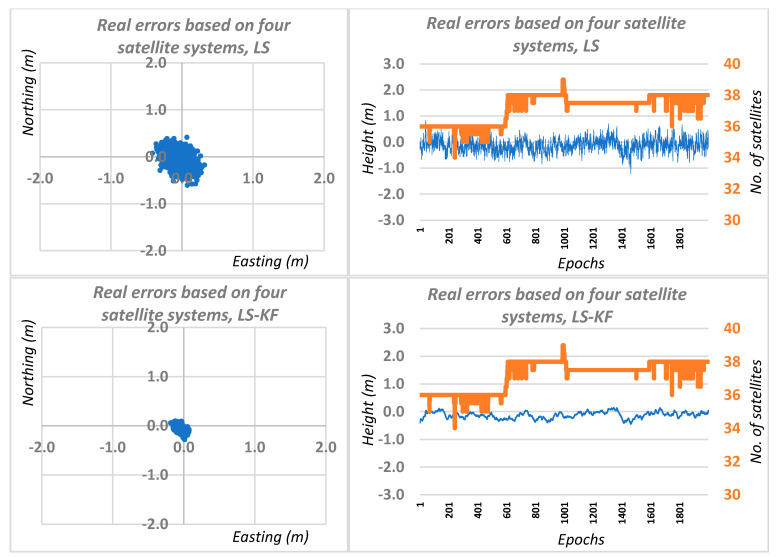
Real errors of the simulated network DGNSS positioning based on least squares solutions and Kalman filtering for four GNSS systems.

Therefore, having two independent observation sessions, we carried out computational simulations, using real errors of DGPS network positioning for two GNSS systems and for four GNSS systems, including the results of DGPS network positioning for sessions I and II. Graphical results of this solution are shown in Figure 6 and Figure 7, respectively, for two and four GNSS systems. Based on Figure 6 and Figure 7, we can see a significant accuracy improvement in relation to traditional DGPS positioning. When using a Kalman filter for the DGNSS network positioning case using two independent positioning systems, the average RMS errors were 0.08, 0.11 and 0.19 m for the N, E, H coordinates, respectively. In the case of the simulation for four DGNSS network positioning systems, the average errors were 0.04, 0.06 and 0.12 m, respectively, for N, E, H coordinates.

## 4. Influence of DD Code Accuracy on Ambiguity Initialization and Methodology of Ambiguity Searching in L1–L5 GPS Data

Although code measurements are of much lower accuracy than precise phase measurements, they are nevertheless necessary in the ambiguity determination process. In the PREFMAR method [34], the determination of the most probable ambiguity sets is performed for the L1 and L5 frequencies, using the following function:

(11) $$\Psi \;{\left(N_{L1}\right)}_{N_{L1}N_{L5}}\;=\;\lambda_{L5}\left({\tilde{N}}_{L5}-{\left[{\tilde{N}}_{L5}\right]}_{\mathrm{roundoff}}\right)\;=\;\;=\;\lambda_{L5}\left(\frac{115\;}{154}(N_{L1}-{\tilde{N}}_{L1,0})+{\tilde{N}}_{L5,0}-{\left[\frac{115}{154}(N_{L1}-{\tilde{N}}_{L1,0})+{\tilde{N}}_{L5,0}\right]}_{\mathrm{roundoff}}\right)$$

where ${\tilde{N}}_{L1,0}$ and ${\tilde{N}}_{L5,0}$ represent a *float* solution or are calculated for a single double difference (DD) measurement as follows:

(12) $${\tilde{N}}_{L1,0}\;=\;\phi_{L1}\;\left(t\right)-\frac{P_{L5\;}\left(t\right)}{\lambda_{L1}}$$

(13) $${\tilde{N}}_{L5,0}\;=\;\phi_{L5}\;\left(t\right)-\frac{P_{L5}\;\left(t\right)}{\lambda_{L5}}$$

We used the function expressed by Formula (11) for a single DD observation, i.e., for phase observations: $\phi_{L1\;}\left(t\right)$, $\phi_{L5\;}\left(t\right)$ and the code observation $P_{L5}\left(t\right).$ However, the observation $P_{L5}\;\left(t\right)$ can be replaced by a more precise pseudo-observation $\rho_{DGNSS}\left(t\right)$, determined from the network DGNSS positioning:

(14) $${\tilde{N}}_{L1,0}\;=\;\phi_{L1}\;\left(t\right)-\frac{\rho_{DGNSS}\left(t\right)}{\lambda_{L1}}$$

(15) $${\tilde{N}}_{L5,0\;}=\;\phi_{L5}\;\left(t\right)-\frac{\rho_{DGNSS}\left(t\right)}{\lambda_{L5}}$$

**Figure 8:**
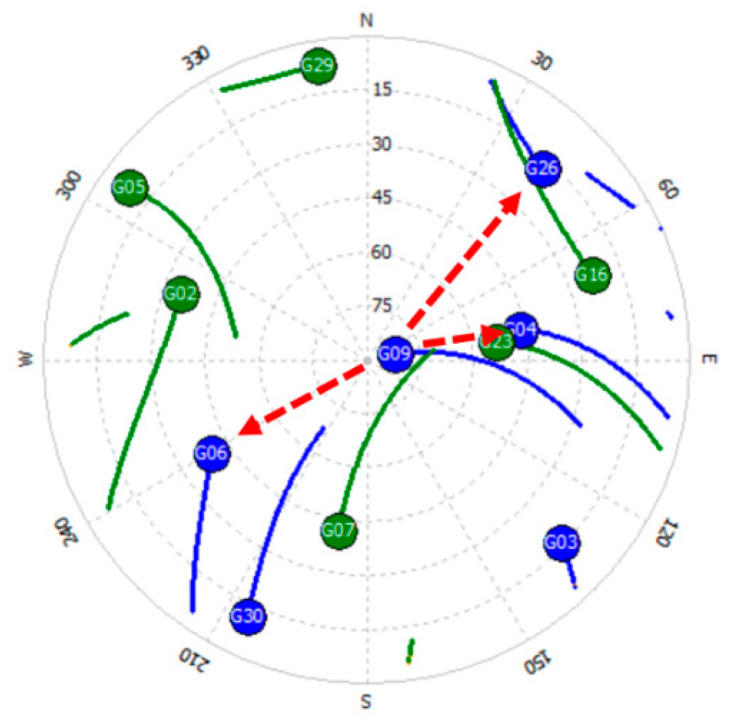
Skyplot of GPS satellites during L1–L5 GPS measurements.

**Figure 9:**
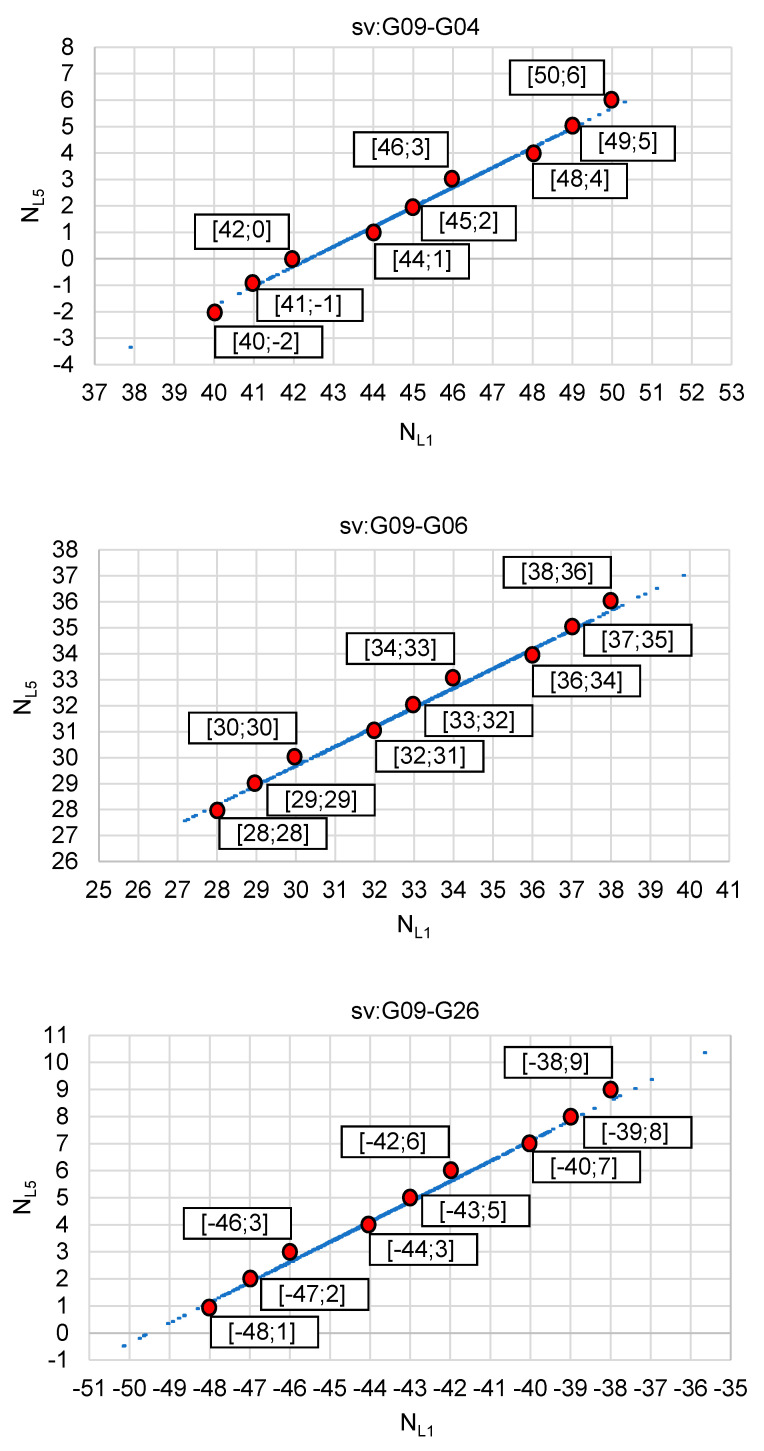
Integer candidates of ambiguity initialization for satellites: G09–G04, G09–G06, G09–G26 if the relative errors of carrier phases L1–L5 are below 10 cm, with the use of the PREFMAR approach.

**Figure 10:**
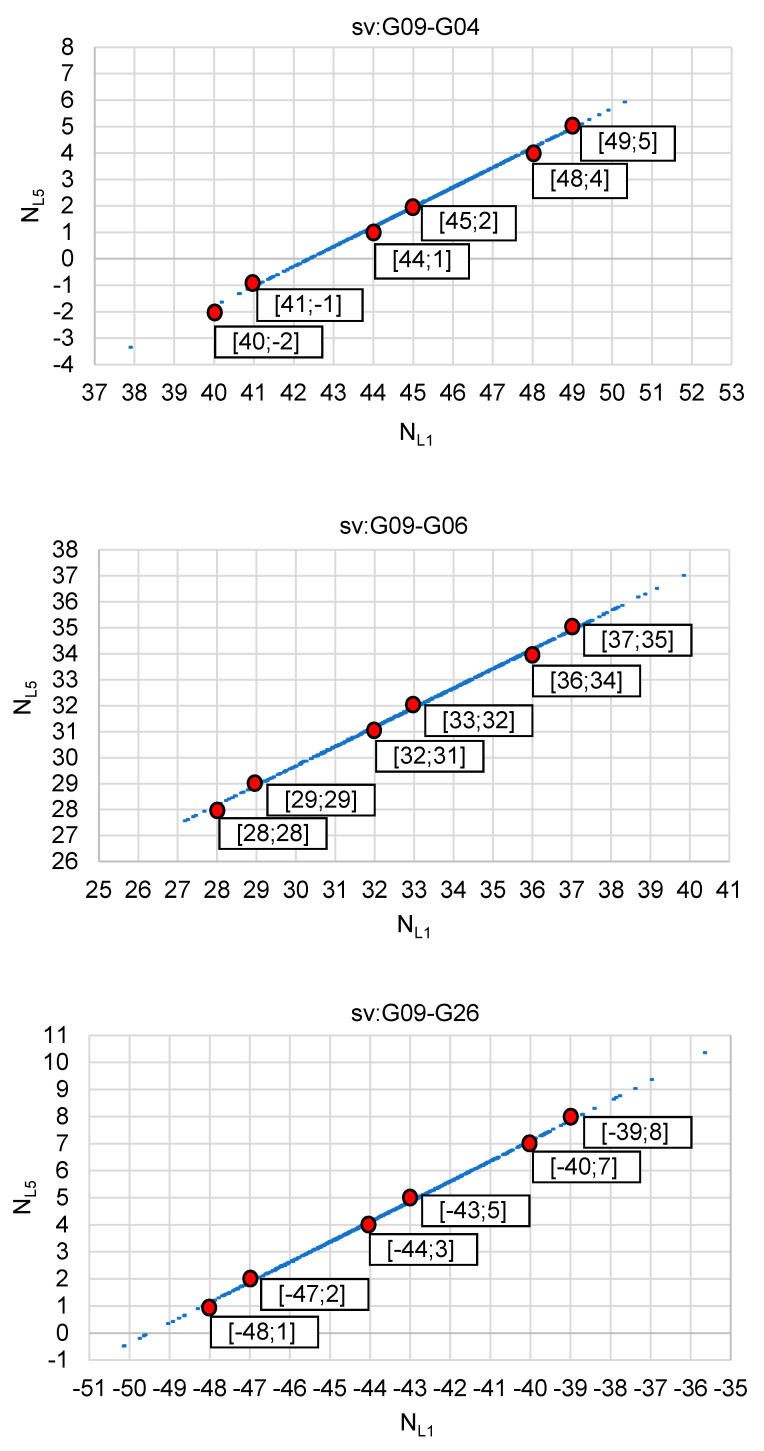
Integer candidates of ambiguity initialization for satellites: G09–G04, G09–G06, G09–G26 if relative errors of carrier phases L1–L5 are below 7 cm, with the use of the PREFMAR approach.

The analysis of such a strategy was tested for real GPS data, for a 4 km baseline. The tests used GPS observations carried out on 22 January 2020 [35], for a ten minute session with an interval of 1 s. The satellite situation during the measurements is shown in Figure 8. The analyses were performed using observations from four GPS satellites: G04, G06, G09 and G26, for which code and phase measurements were recorded at the frequencies L1 = 1575.42 MHz and L5 = 1176.45 MHz. Solutions of float ambiguities, determined only for single DD observations from two satellites, based on Formulas (12) and (13) are shown in Figure 9 and Figure 10. Additionally, these figures show the sets of the most probable ambiguities N1–N5 depending on the assumed relative errors of the L1 and L5 phase observations, using the function used in the PREFMAR method and expressed by Formula (11).

Figure 9 shows possible ambiguities for DD observations and satellite pairs: G09–G04, G09–G06, G09–G26, assuming that the relative errors of phase observations do not exceed 10 cm. It is therefore quite a large value, suitable for baselines with a length of even several or several dozen kilometers, depending on the observation conditions, i.e., depending on the activity of the ionosphere or depending on the position of the satellites above the horizon. In the case of the use of the three reference stations available, there is no need to determine long baselines between the mobile receiver and any of these reference stations. We only determine the baseline with respect to the virtual reference station. As is known, a reference station can be created at any position, e.g., at a distance of several meters, with respect to our 3D position. In such cases, we can therefore assume that the relative errors of phase observations do not exceed, for example, 7 cm. This immediately results in a reduction of the number of possible ambiguity sets that can potentially be searched unknowns (Figure 10). Thus, for relative errors smaller than 7 cm, based on the P (L5) code observations only, we have for each pair of satellites six ambiguity pairs for validation, which for three DD observations gives 6^3^ = 216 combinations. Therefore, quite a lot of combinations as for RTK measurements. However, if we increase the accuracy of DD code observations to 0.47 m, we will reduce the number of potential ambiguities to three sets for each pair of satellites, and this for relative errors up to 7 cm. Thus, for three independent DD observations, we get 3^3^ = 27. If we increase even more the accuracy of the code measurements to 0.38 m, then for relative errors less than 7 cm, we get only 2^3^ = 8 combinations for four satellites. Such a small number of combinations will significantly reduce the initialization time of the determined ambiguities. It should also be noted that for code errors below 0.38 cm and for relative errors of phase observations below 3 cm, we are able to unambiguously determine the searching ambiguity sets for a given DD observation [34]. In practice, for RTK measurements, we should safely use the range (−7 cm; +7 cm), which should be sufficient for RTK positioning using virtual reference stations, or for baselines in fast static measurements, with lengths of about a few kilometers. Furthermore, it is also safe to use the N1–N5 ambiguity search range for L1–L5 frequencies below 0.47 m; which results in obtaining 27 combinations for relative errors of up to 7 cm for three independent DD phase observations obtained from individual measurement epochs.

The presented analysis of DGNSS network positioning accuracy indicates that obtaining network positioning accuracy below 0.47 m is already possible with the use of only the GPS system and the civil code P (L1). However, for the full structure of the GPS signal, that is, the L5 frequency, there will be the possibility of a greater use of the P (L5) code which will further increase the accuracy of the DGNSS network positioning.

Thus, we can see how important code measurements are in the ambiguity initialization process. Therefore, the use of a DGNSS network solution will significantly accelerate the ambiguity determination, both in static and kinematic measurements. It should also be noted that in this methodology, for determining ambiguity sets, based on the function expressed by Equation (11), we need only single phase observations of the second differences, without the need to determine the approximate ambiguities of the float type. Moreover, it is not necessary that the DD phase observations for the satellite pairs: G09–G04, G09–G06 and G09–G26 be at the same time (*t*). Each observation may be from a different observation epoch, furthermore, we only need one DD epoch to indicate the probable ambiguity sets for validation. Moreover, using the function expressed by Equations (11) with (14) and (15), we were also able to indicate the ambiguity sets for the remaining combinations of satellite pairs: G06–G26; G26–G04 and G06–G06. Such a solution can be effective for precise positioning with the use of smartphones. This is related to the selection of the reference satellite when determining DD observations, i.e., GNSS antennas in mobile phones have much worse access to satellites than traditional antennas in GNSS receivers, especially when the smartphone is in a vertical position above the observed point, while reference satellites are selected as those that are at the zenith [35]. While for geodetic receivers it is an optimal solution when the reference satellite is in zenith, and for precise phase positioning with the use of mobile phones, it is a big problem, because the reference satellite located at the zenith, due to the housing of the cell phone, will have a significantly worse signal than the satellites lower above the horizon if the phone is oriented vertically during the measurement.

## 5. Discussion

The accuracy and reliability of GNSS positioning depends on several major factors, such as the type of equipment (hardware), software (algorithms used), measurement technology and processing GNSS data. Each of these elements is crucial. To obtain accurate and reliable coordinates, especially for precise navigation purposes, we must use both the appropriate class of GNSS satellite receivers as well as know the capabilities of the used algorithms. Only then should we choose the appropriate measurement and validation technology for the coordinates being determined. Therefore, GNSS positioning is quite complicated, and requires from a user both theoretical and practical knowledge of GNSS positioning techniques, as well as knowledge of the characteristics of various errors occurring in satellite observations. One of the ways for a reliable GNSS navigation is the use of several GNSS systems independently, e.g., GPS, GLONASS, Galileo and BeiDou. The use of such a number of satellites results in a significant increase in DGNSS positioning accuracy from about 1–2 m to about 0.1–0.2 m, using Kalman filtering. Reliability in aerial navigation plays an important role. Therefore, it can be obtained by using at least two or four independent GNSS navigation systems. Based on the conducted research, it can be concluded that obtaining 3D accuracy at a level below 0.47 m using only code observations and a network solution is possible with the use of only one GNSS system. Such accuracy already causes a significant reduction in the probable sets of ambiguities for relative errors of the L1–L5 phase observations from the interval (−7 cm; +7 cm). Such accuracy of the DGPS network positioning means that for four satellites and three independent DD observations, we have 3^3^ = 27 combinations in the ambiguity initialization process.

In the case of network DGNSS positioning, using only two independent satellite systems, it is possible to obtain an accuracy below 0.4 m, which immediately reduces the possible ambiguity sets up to two for each pair of satellites. Which for four satellites gives us 2^3^ = 8 independent combinations, that is, reducing all combinations from 216 to 8. Such accuracy of the GNSS network code positioning will therefore enable much faster ambiguity determination for L1–L5 phase measurements for a single measurement epoch.

## 6. Conclusions

This paper presents the possibilities of GNSS network positioning in the case of one, two and four independent DGNSS reference station positioning systems. The calculations were made using code measurements on the L1 frequency. The research was carried out in the scope of DGNSS code technology accuracy using GPS system and permanent reference stations of the ASG-EUPOS system.

While the measurement accuracies of code observations (meters) are significantly inferior to the measurement accuracy of phase observations (millimeters), the importance of code observations is fundamental for all GNSS systems. Without code measurements, there would be no GNSS navigation and there would be no possibility of precise phase positioning. Therefore, in this work, computational simulations were carried out based on real GPS observations, for the case of using two or four independent and equivalent GNSS positioning systems.

On the basis of real DGPS network positioning tests performed using only the GPS system, the average RMS errors were obtained at the level of 3 decimeters for the horizontal position and at the level of 6 decimeters for the vertical position, but only using the least squares method. However, in the case of using additional Kalman filtering, the average RMS errors were obtained at the level of 15 cm for the horizontal coordinates and at the level of 30 cm for the vertical coordinate.

The simulation tests were conducted on the basis of GPS observations only. Therefore, it was assumed that all current independent GNSS systems (e.g., GPS, GLONASS, Galileo or BeiDou) have similar positioning accuracy. Therefore, such simulations can be performed, which largely make it possible to evaluate the integration of any number of independent satellite positioning systems. Based on the conducted computational simulations, in which scenarios for two and four different GNSS systems were created, a significant increase in accuracy was obtained. For two systems, the average RMS errors were 0.08, 0.11 and 0.19 m for the N, E, H coordinates. In the case of simulation for four DGNSS network positioning systems, the average errors were 0.04, 0.06 and 0.12 m, respectively for the N, E, H coordinates. Such accuracy allows instantaneous ambiguity initialization using PREFMAR and other methods.

## Figures and Tables

**Table 1:** Statistical characteristics of the DGPS network positioning results for session I (meters).

Solution	RMS (E); LS	RMS (N); LS	RMS (H); LS	RMS (E); LS–KF	RMS (N); LS–KF	RMS (H); LS–KF
ELBL-M	0.243	0.321	0.579	0.112	0.137	0.235
ILAW-M	0.263	0.338	0.670	0.112	0.137	0.235
KROL-M	0.324	0.432	0.779	0.177	0.217	0.395
OLST-M	0.444	0.702	1.109	0.282	0.462	0.654
NETWORK	0.232	0.313	0.562	0.131	0.167	0.299

**Table 2:** Statistical characteristics of DGPS network positioning results for session II.

Solution	RMS (E); LS	RMS (N); LS	RMS (H); LS	RMS (E), LS–KF	RMS (N); LS–KF	RMS (H); LS–KF
ELBL-M	0.239	0.337	0.583	0.101	0.152	0.282
ILAW-M	0.251	0.333	0.610	0.116	0.138	0.300
KROL-M	0.296	0.416	0.703	0.126	0.179	0.288
OLST-M	0.423	0.662	1.108	0.249	0.408	0.649
NETWORK	0.217	0.304	0.523	0.100	0.135	0.241
